# Ab interno trabeculotomy with vs. without cataract surgery for POAG: a retrospective study

**DOI:** 10.3389/fmed.2026.1914639

**Published:** 2026-09-03

**Authors:** Lei Zhang, Junjie Bian

**Affiliations:** Department of Ophthalmology, Xuanwu Hospital Capital Medical University, Bei Jing, China

**Keywords:** IOP spikes, phacoemulsification, primary open-angle glaucoma, surgery, trabeculotomy

## Abstract

**Purpose:**

To compare the 12-month efficacy and safety of ab interno trabeculotomy combined with phacoemulsification vs. ab interno trabeculotomy alone in patients with primary open-angle glaucoma(POAG).

**Methods:**

A retrospective analysis was performed on clinical data from patients diagnosed with POAG between January 2021 and January 2025 who underwent either combined surgery (combined group, 39 cases)or ab interno trabeculotomy alone (ab interno trabeculotomy-only group,44 cases). The primary outcome measures were postoperative intraocular pressure (IOP) and the change in IOP from baseline. Secondary outcomes included the number of antiglaucoma medications, surgical success rates (complete success and qualified success), and the incidence of complications (hyphema and IOP spike).

**Results:**

Baseline characteristics were comparable between the two groups. At postoperative day 1, week 1, months 1, 3, and 6, IOP in the combined group was significantly lower than that in the ab interno trabeculotomy-only group (all *P* < 0.05). At 12 months postoperatively, no statistically significant difference in IOP was observed between the groups (15.94 ± 1.45 mmHg vs. 16.15 ± 1.36 mmHg, *P* = 0.266). The magnitude of IOP reduction was greater in the combined group than in the ab interno trabeculotomy-only group at all time points (all *P* < 0.05). No statistically significant differences were found between the two groups regarding the number of antiglaucoma medications or the reduction in medication use at any postoperative time point (all *P* > 0.05). At 12 months postoperatively, neither complete nor qualified success rates differed significantly between the groups. The incidence of hyphema was lower in the combined group than in the ab interno trabeculotomy-only group (23.08% vs. 43.18%, *P* = 0.053), and the incidence of IOP spike was significantly lower in the combined group (12.82% vs. 38.63%, *P* = 0.005).

**Conclusion:**

In patients with POAG, combined ab interno trabeculotomy with phacoemulsification achieved superior IOP reduction and a significantly lower IOP spike rate compared with trabeculotomy alone. However, no statistically significant differences were observed at medication reduction, or surgical success rates. Given the retrospective, non-randomized design and baseline imbalances, the combined procedure cannot be definitively recommended as the preferred approach. These findings should be considered hypothesis-generating, and prospective randomized trials are warranted to validate them.

## Introduction

Glaucoma is the leading cause of irreversible blindness globally. It is a chronic, progressive optic neuropathy, and the number of affected individuals is projected to reach 111.8 million by 2040, with primary open-angle glaucoma being the most prevalent subtype (1). The estimated prevalence among individuals aged 40–80 years is 3.0% (2). Risk factors for open-angle glaucoma include older age, African ancestry or Latino/Hispanic ethnicity, elevated intraocular pressure, a family history of glaucoma, low ocular perfusion pressure, type 2 diabetes, and thin central cornea (3). Timely early diagnosis and effective treatment can prevent blindness. POAG is a progressive optic neuropathy characterized by an open anterior chamber angle, elevated intraocular pressure, characteristic optic disc changes, and corresponding visual field defects. The primary pathogenic factor is elevated intraocular pressure, and the main mechanism underlying this elevation is increased aqueous humor outflow resistance (4). The current core objective of POAG treatment is to lower intraocular pressure (IOP) to target levels, thereby slowing or halting disease progression (5).

Glaucoma surgical treatments have been continuously evolving. Trabeculectomy remains the classic surgical approach for glaucoma management. As currently defined, ab interno trabeculotomy is classified as a minimally invasive glaucoma surgery (MIGS). It offers the advantages of short operative time, minimal trauma, rapid postoperative recovery, fewer complications, and effective IOP reduction. By directly creating a channel between the anterior chamber and Schlemm's canal, it enhances aqueous humor outflow via the Schlemm's canal pathway, thereby lowering IOP. This procedure is particularly suitable for patients who have failed or are intolerant to maximally tolerated medical therapy. However, stand-alone ab interno trabeculotomy is associated with a relatively high rate of postoperative IOP spike. Cataract surgery, especially phacoemulsification with intraocular lens (IOL) implantation, has been demonstrated to produce a modest but definite IOP-lowering effect in both glaucomatous and non-glaucomatous patients.The proposed mechanisms may include stretching of the trabecular meshwork, increased uveoscleral outflow, and reduction of inflammatory mediators. Combining ab interno trabeculotomy with cataract surgery offers the potential advantage of addressing both lens opacity and glaucoma in a single procedure, thereby reducing the need for multiple surgeries, lowering IOP, and potentially reducing the incidence of postoperative IOP spike.

In this retrospective study, we compared the 12-month outcomes of phacoab interno trabeculotomy vs. stand-alone ab interno trabeculotomy for the treatment of POAG. The two groups were compared in terms of postoperative IOP, use of IOP-lowering medications, surgical success rate, and postoperative complications.

## Methods

### Study design

This was a retrospective study involving data collected from glaucoma patients treated at Xuanwu Hospital, Capital Medical University, between January 2021 and January 2025. The study adhered to the tenets of the Declaration of Helsinki and was approved by the Ethics Committee of Xuanwu Hospital, Capital Medical University(No.MR-11-25-033798). Due to the retrospective nature of the study, the Ethics Committee waived the requirement for informed consent. All patient data were anonymized and contained no personally identifiable information.

### Patients

We retrospectively reviewed the medical records of patients diagnosed with POAG who underwent combined surgery (combined group) or stand-alone ab interno trabeculotomy (stand-alone ab interno trabeculotomy group). Briefly, all enrolled patients had valid indications for glaucoma surgery. The decision to perform combined phacoemulsification was primarily based on the following factors: the presence of clinically significant cataracts with lens opacity leading to visual impairment, the surgeon's clinical judgment that combined surgery was feasible, and the patient's own willingness to undergo cataract surgery. For patients in the ab interno trabeculotomy-alone group, the reasons were twofold: some patients had only mild cataracts that did not meet the surgical indications, whereas others had cataracts that clinically met the indications but declined cataract surgery and specifically chose to proceed with glaucoma surgery alone.

 Inclusion criteria: Diagnosed with POAG, POAG diagnostic criteria: pathological elevated intraocular pressure, 24-hour peak IOPexceeding 21 mmHg; anterior chamber angle remains open during IOP elevation; presence of acquired glaucomatous characteristic retinal/optic nerve damage and/or visual field damage; visual field damage is mild to moderate; no causative ocular or systemic abnormalities associated with elevated intraocular pressure (6); age ≥55 years; had undergone combined cataract and ab interno trabeculotomy surgery or stand-alone ab interno trabeculotomy surgery; cataract type before surgery in the combined group was senile cataract; complete 12-month postoperative follow-up data available; the stand-alone ab interno trabeculotomy group did not undergo cataract surgery again within 12 months postoperatively; all patients had not undergone any other ocular surgery except the surgery related to this study.

Exclusion criteria: Secondary glaucoma (pseudoexfoliation, pigment dispersion, uveitic, neovascular, or angle-closure glaucoma); prior history of incisional glaucoma surgery or cyclodestructive procedures; history of ocular trauma with angle recession; history of ocular trauma, corneal opacity affecting gonioscopy or IOP measurement; diseases that may affect the accuracy of IOP measurement, such as orbital space-occupying lesions, thyroid-associated ophthalmopathy.

### Surgical methods

All surgeries were performed by the same senior glaucoma specialist.

**Figure 1:**
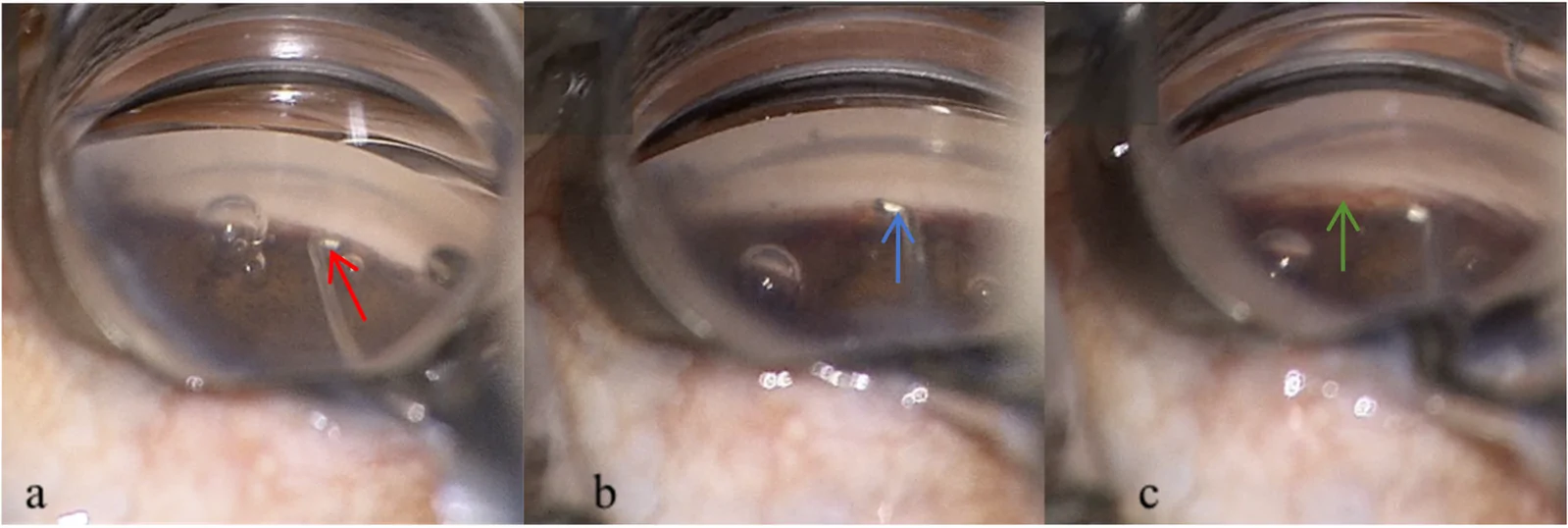
Ab interno ntrabeculotomy procedure. **(a)** A trabeculotome is used to incise approximately 60° of the trabecular meshwork in the direction of the knife tip. The red arrow indicates the tip of the trabeculotome. **(b)** The direction of the trabeculotome is reversed, and another incision of approximately 60° of the trabecular meshwork is made in the direction of the knife tip. The blue arrow indicates the tip of the trabeculotome. **(c)** The green arrow points to the incised trabecular meshwork, exposing the underlying white sclera. Owing to the retrospective design of this study and the limited availability of imaging data, the image clarity is suboptimal.

Combined surgery group: A clear corneal main incision was made at the temporal limbus, and an auxiliary side port incision was made at the 2 o'clock position. Viscoelastic material was injected into the anterior chamber to maintain chamber depth. Under direct gonioscopic visualization, a trabeculotome was used to incise the nasal trabecular meshwork for approximately 120°, taking care to avoid injury to the iris root and peripheral cornea. After completing the ab interno trabeculotomy, continuous curvilinear capsulorhexis (approximately 5.0–5.5 mm in diameter) was performed. The lens nucleus was phacoemulsified using a chopping technique, and residual cortex was aspirated. An IOL was implanted in the capsular bag. Following IOL implantation, the irrigation/aspiration system of the phacoemulsification machine was used to carefully aspirate any residual blood, viscoelastic material, and cortical fragments from the anterior chamber. After confirming stable anterior chamber formation, the main and side port incisions were closed in a watertight manner.ab interno trabeculotomy-only group: The incision steps were the same as above. Viscoelastic material was injected into the anterior chamber, and under direct gonioscopic visualization, the same trabeculotome was used to incise the nasal trabecular meshwork for approximately 120° (Figure 1). A syringe filled with normal saline connected to an irrigation cannula was used to repeatedly irrigate the anterior chamber to remove residual viscoelastic material and blood. The irrigation pressure was adjusted to produce only mild aqueous fluctuation, avoiding violent surging of the anterior chamber. The main and side port incisions were closed in a watertight manner.

### Outcome measures

Primary outcome measure: Intraocular pressure. Secondary outcome measures:Number of anti-glaucoma medications used at each follow-up visit.Surgical success: Postoperative IOP decreased by ≥20% compared to baseline, and no increase in glaucoma medications compared to preoperatively, and no additional anti-glaucoma surgery required.Qualified success: IOPdecreased compared to baseline, and the number of glaucoma medications reduced by at least 1, and no additional anti-glaucoma surgery required. The reduction in glaucoma medication was calculated starting from 1 months postoperatively. Complications: Hyphema (visible layered blood in the anterior chamber); IOP spike (defined as any postoperative IOPelevation ≥10 mmHg compared to preoperative within 3 months postoperatively). During the 12-month follow-up period, patients with surgical failure and high IOP judged to require further intraocular pressure-lowering surgery underwent other glaucoma surgeries, with the surgical options being trabeculectomy or glaucoma drainage device implantation.

### Statistical analysis

Continuous variables (IOP, number of medications, BCVA) were assessed for normality using the Shapiro–Wilk test. Normally distributed data were expressed as mean, and comparisons between groups were performed using the independent samples t-test, while within-group comparisons were performed using the paired t-test. Non-normally distributed data were analyzed using the Mann–Whitney *U* test or Wilcoxon signed-rank test. Categorical variables (success rate, complication rate) were compared using the chi-square test or Fisher's exact test. *P* < 0.05 was considered statistically significant. Cumulative success curves up to 12 months postoperatively were constructed using the Kaplan–Meier method to conduct survival analysis. Failure events were defined as not meeting the criteria for either complete or qualified success as described above. Data for patients who had not experienced a failure event by the final follow-up were treated as censored. The log-rank test was used to compare the differences in cumulative success rates between the two groups. All analyses were performed using SPSS 26.0 software.

## Results

### Baseline characteristics

Baseline demographic and clinical characteristics are summarized in Table 1. There were no statistically significant differences between the two groups in age (*P* = 0.745), gender distribution (*P* = 0.514), POAG severity (*P* = 0.375), baseline IOP (*P* = 0.095), number of glaucoma medications (*P* = 0.152), central corneal thickness (*P* = 0.671), or MD (*P* = 0.598). All patients in the combined group had cataract, whereas 27 of the 44 eyes (61.4%) in the trabeculotomy-alone group had cataract. Compared with the trabeculotomy-alone group, the combined group had higher LOCS III nuclear opalescence grades (median 4.50 vs. 2.00), and the difference was statistically significant (*P* < 0.001). Similarly, the combined group had worse BCVA (median LogMAR 0.68 vs. 0.32), and the difference was also statistically significant (*P* < 0.001).

**Table 1:** Baseline demographic and clinical characteristics of patients in the two groups.

Variables	Combined group	Ab Interno Trabeculotomy alone group	*P-*value
Age (y)	69.15 ± 4.83	67.78 ± 4.71	0.745
Gender (male/female)	23/16	29/15	–
No. of eyes (no. of patients)	39 (39)	44 (44)	–
POAG severity, *n* (%)	0.375		
Mild	17 (43.6)	15 (34.1)	–
Moderate	22 (56.4)	29 (65.9)	–
Severe	0	0	–
Cataract presence, *n* (%)	39 (100)	27 (61.4)	–
LOCS III Nuclear Opalescence (NO), median (Q1, Q3)	4.50 (3.80, 5.20)	2.00 (1.50, 3.00)	<0.001
BCVA (LogMAR), median (Q1, Q3)	0.68 (0.52, 1.02)	0.32 (0.21, 0.53)	<0.001
IOP (mmHg)	23.13 ± 3.16	22.29 ± 2.54	0.095
Number of glaucoma medicines (*n*)	3.54 ± 0.64	3.73 ± 0.62	0.161
CCT(μm)	549.44 ± 52.57	540.35 ± 24.54	0.671
MD(db)	−6.11 ± 3.01	−6.70 ± 3.13	0.610

POAG, primary open-angle glaucoma; IOP, intraocular pressure; MD, mean deviation; CCT, central corneal thickness; BCVA, best corrected visual acuity.

### Intraocular pressure

**Figure 2:**
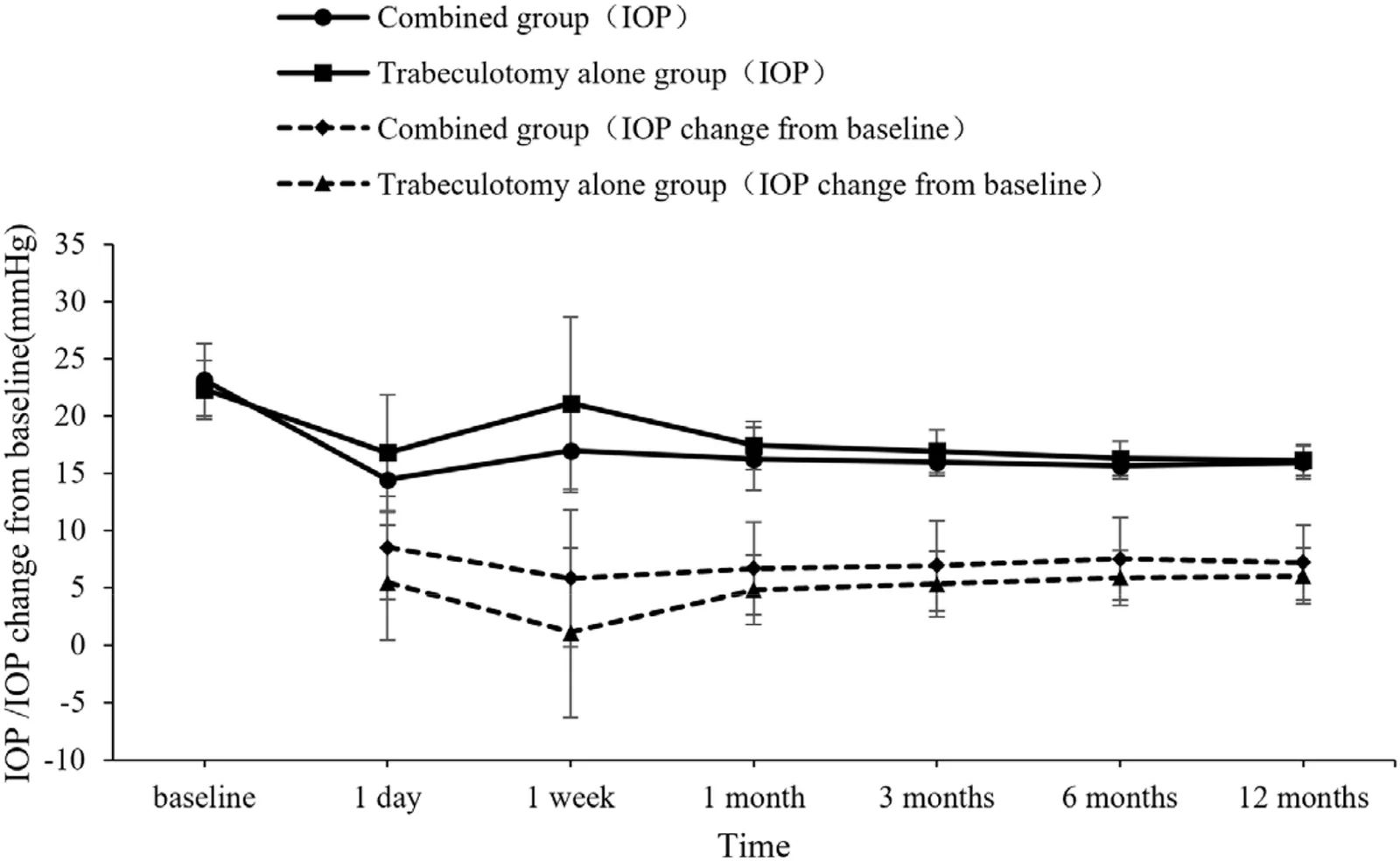
Intraocular pressure in the two groups of patients, IOP, intraocular pressure.

Table 2 summarizes the IOP and its change from baseline at each postoperative follow-up time point in the two groups. At postoperative day 1, week 1, months 1, 3, and 6, IOP in the combined group was significantly lower than that in the ab interno trabeculotomy-alone group (14.42 ± 2.81 mmHg vs. 16.79 ± 5.06 mmHg, *P* = 0.012; 16.98 ± 3.64 mmHg vs. 21.12 ± 7.54 mmHg, *P* = 0.005; 16.23 ± 2.74 mmHg vs. 17.43 ± 2.08 mmHg, *P* = 0.034; 15.97 ± 1.21 mmHg vs. 16.92 ± 1.85 mmHg, *P* = 0.009; 15.62 ± 1.08 mmHg vs. 16.30 ± 1.50 mmHg, *P* = 0.014, respectively). At 12 months postoperatively, IOP remained lower in the combined group than in the ab interno trabeculotomy-alone group (15.94 ± 1.45 mmHg vs. 16.15 ± 1.36 mmHg), but the difference was not statistically significant (*P* = 0.266). Regarding the magnitude of IOP reduction, the combined group exhibited a greater reduction than the ab interno trabeculotomy-alone group at all time points. The differences in IOP reduction between the two groups were statistically significant at postoperative day 1, week 1, months 1, 3, 6, and 12 (*P* = 0.005, 0.004, 0.026, 0.039, 0.012, and 0.045, respectively) (Figure 2).

**Table 2:** Intraocular pressure in the two groups of patients.

Variables	Combined group (*n* = 39)	Ab interno trabeculotomy alone group(*n* = 44)	*P-*value
IOP (mmHg)			
Baseline	23.13 ± 3.16	22.29 ± 2.54	0.095
1 d after surgery	14.42 ± 2.81	16.79 ± 5.06	0.012
1 w after surgery	16.98 ± 3.64	21.12 ± 7.54	0.005
1 m after surgery	16.23 ± 2.74	17.43 ± 2.08	0.034
3 m after surgery	15.97 ± 1.21	16.92 ± 1.85	0.009
6 m after surgery	15.62 ± 1.08	16.30 ± 1.50	0.014
12 m after surgery	15.94 ± 1.45	16.15 ± 1.36	0.266
IOP change from baseline (mmHg)			
1 d after surgery	8.50 ± 4.51	5.43 ± 4.99	0.005
1 w after surgery	5.80 ± 5.96	1.10 ± 7.38	0.004
1 m after surgery	6.66 ± 4.04	4.81 ± 3.02	0.026
3 m after surgery	6.94 ± 3.93	5.31 ± 2.88	0.039
6 m after surgery	7.51 ± 3.58	5.87 ± 2.40	0.012
12 m after surgery	7.19 ± 3.26	6.01 ± 2.46	0.045

IOP, intraocular pressure.

### Glaucoma medications

**Figure 3:**
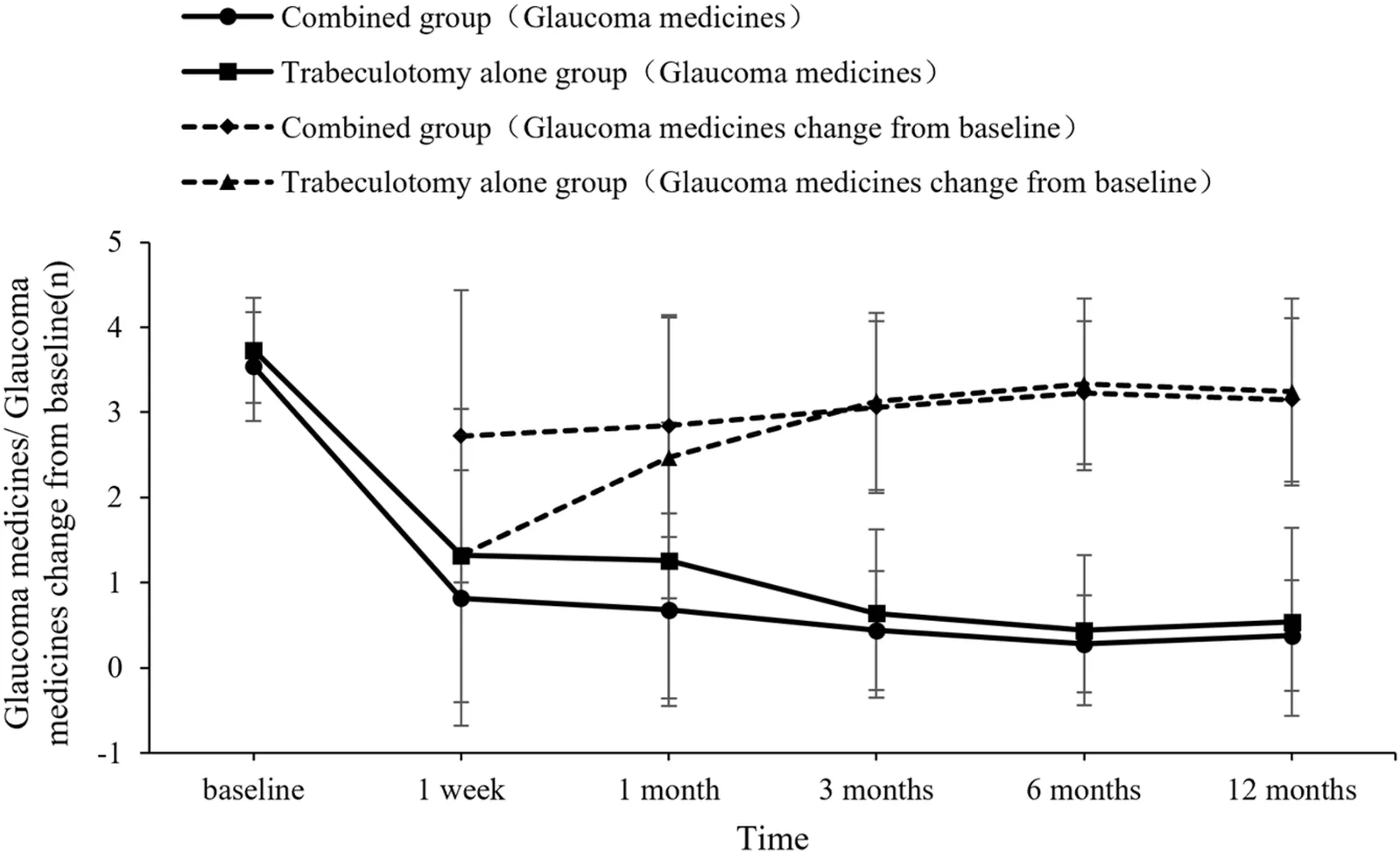
Use of glaucoma medications in the two groups of patients.

The number of antiglaucoma medications at baseline was similar between the two groups (combined group: 3.54 ± 0.64; ab interno trabeculotomy-alone group: 3.73 ± 0.62; *P* = 0.161). At postoperative week 1, months 1, 3, 6, and 12, the number of medications in the combined group was 0.82 ± 1.50, 0.68 ± 1.13, 0.44 ± 0.70, 0.28 ± 0.57, and 0.38 ± 0.65, respectively; in the ab interno trabeculotomy-alone group, the corresponding values were 1.32 ± 1.72, 1.26 ± 1.62, 0.64 ± 0.99, 0.44 ± 0.88, and 0.54 ± 1.10. Although the combined group had lower medication counts at all time points than the ab interno trabeculotomy-alone group, the differences between the groups were not statistically significant (all *P* > 0.05). The changes in the number of medications from baseline also showed no significant differences between the two groups at any time point (all *P* > 0.05) (Table 3) (Figure 3).

**Table 3:** Use of glaucoma medications in the two groups of patients.

Variables	Combined group (*n* = 39)	Ab Interno Trabeculotomy alone group(*n* = 44)	*P-*value
Glaucoma medicines (*n*)			
Baseline	3.54 ± 0.64	3.73 ± 0.62	0.161
1 w after surgery	0.82 ± 1.50	1.32 ± 1.72	0.167
1 m after surgery	0.68 ± 1.13	1.26 ± 1.62	0.071
3 m after surgery	0.44 ± 0.70	0.64 ± 0.99	0.325
6 m after surgery	0.28 ± 0.57	0.44 ± 0.88	0.363
12 m after surgery	0.38 ± 0.65	0.54 ± 1.10	0.451
Glaucoma medicines change from baseline (*n*)			
1 w after surgery	2.72 ± 1.72	1.32 ± 1.72	0.427
1 m after surgery	2.84 ± 1.30	2.47 ± 1.65	0.272
3 m after surgery	3.06 ± 1.01	3.13 ± 1.04	0.770
6 m after surgery	3.23 ± 0.84	3.33 ± 1.01	0.632
12 m after surgery	3.15 ± 0.96	3.24 ± 1.10	0.715

### Surgical success rate

**Figure 4:**
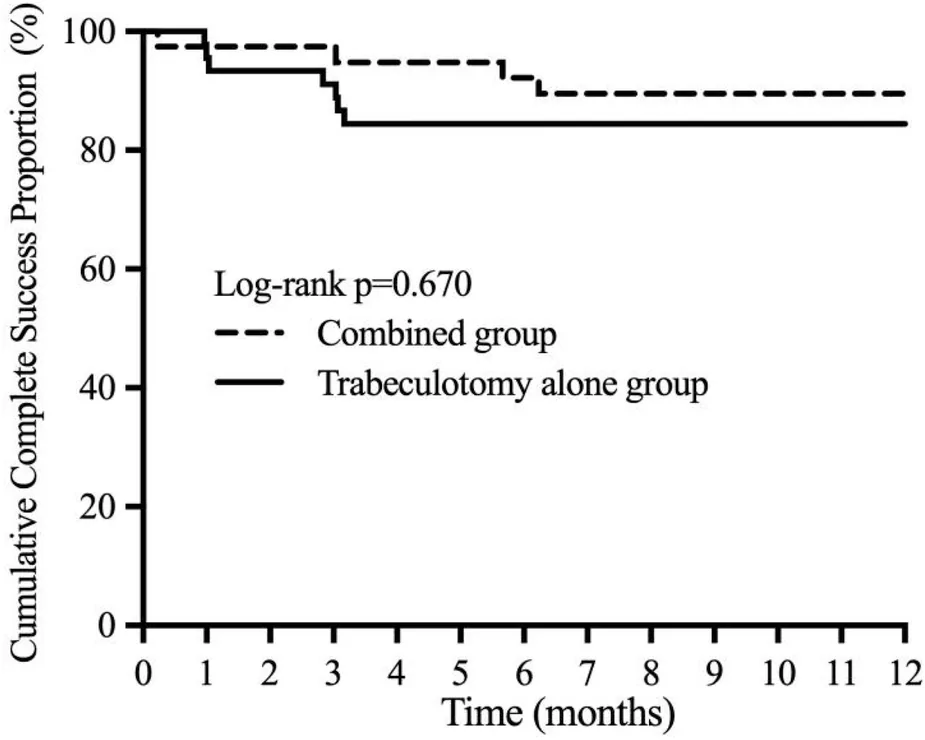
Cumulative complete success rate after surgery in the two patient groups.

**Figure 5:**
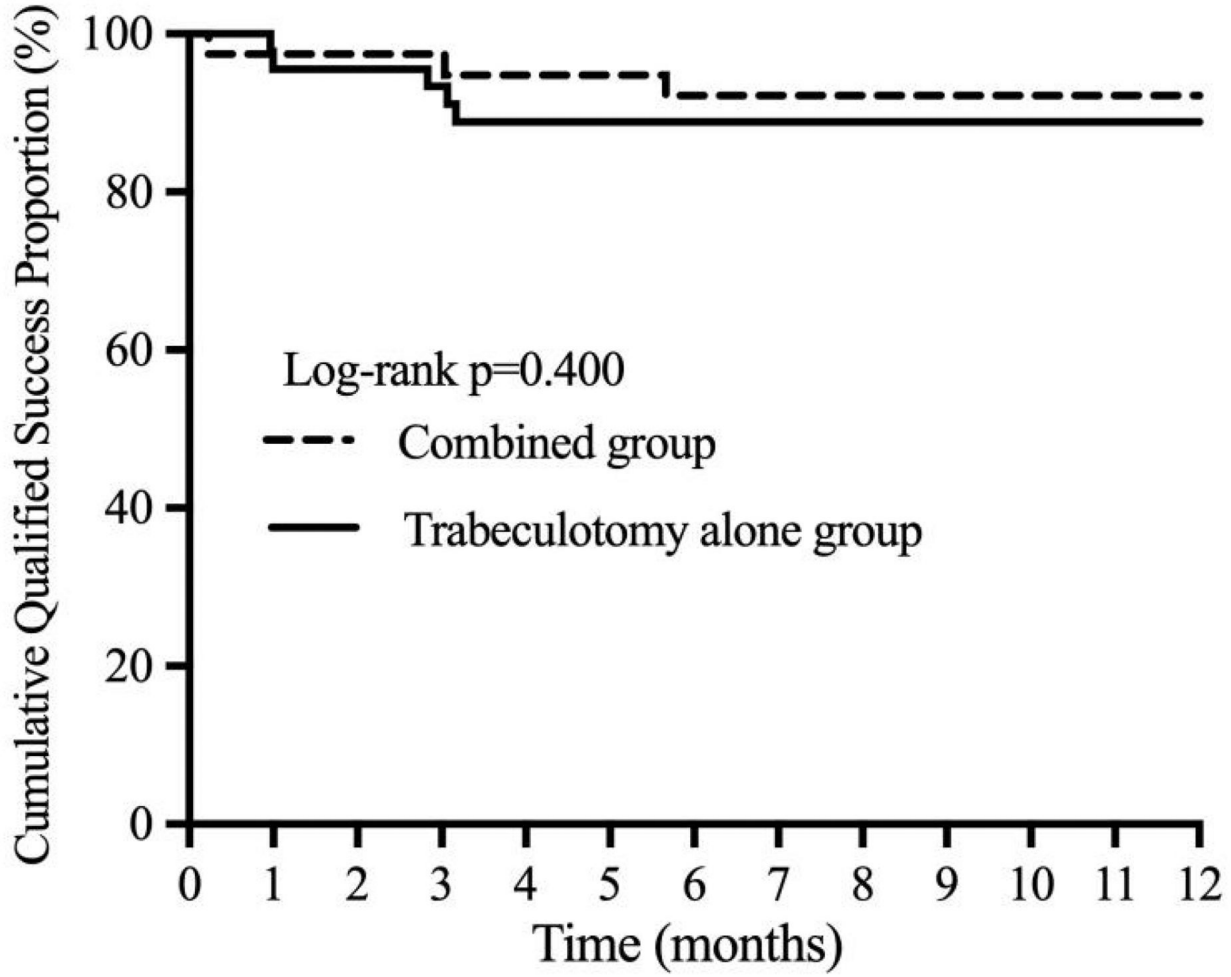
Cumulative conditional success rate after surgery in the two patient groups.

At 12 months postoperatively, the complete success rate was 84.62% (33/39) in the combined group and 77.27% (34/44) in the ab interno trabeculotomy-alone group, with no statistically significant difference (*P* = 0.397, *χ*^2^ test). The qualified success rate was 89.74% (35/39) in the combined group and 84.09% (37/44) in the ab interno trabeculotomy-alone group, also showing no statistically significant difference (*P* = 0.448, *χ*^2^ test). To further evaluate the time-to-event distributions for achieving success criteria, cumulative success rates were estimated using the Kaplan–Meier method, and the corresponding survival curves are presented in Figures 4, 5; patients who did not experience a failure event were censored at the last follow-up. The log-rank test was used to compare the cumulative success rates between the two groups, revealing no statistically significant difference for complete success (*P* = 0.670) or for qualified success (*P* = 0.400).

**Figure 6:**
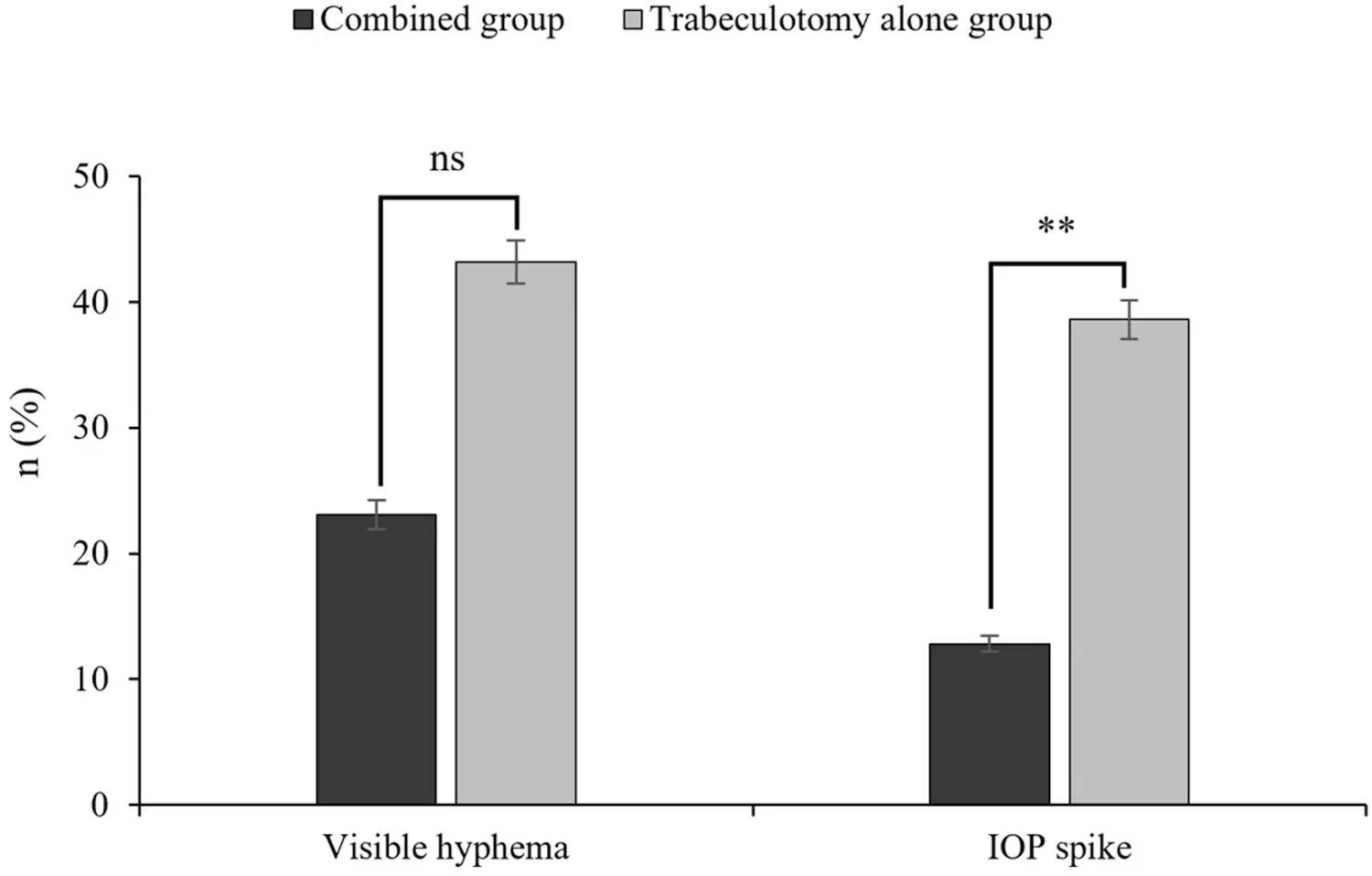
Postoperative complications; IOP, intraocular pressure; **, *p* = 0.005.

### Complications

The incidence of visible hyphema was 23.08% (9/39) in the combined group and 43.18% (19/44) in the ab interno trabeculotomy group, with no significant difference (*P* = 0.053). The incidence of IOP spike was significantly lower in the combined group (12.82%, 5/39) than in the ab interno trabeculotomy group (38.63%, 17/44; *P* = 0.005). For management and outcomes, all 5 cases in the combined group were treated with anterior chamber paracentesis and IOP-lowering medications, and achieved normal IOP within 2 weeks. Among the 17 cases in the ab interno trabeculotomy group, 1 underwent anterior chamber washout, 13 received paracentesis plus medications and recovered within 2 weeks; the remaining 3 cases had persistent IOP spike and subsequently underwent trabeculectomy (Table 4) (Figure 6).

**Table 4:** Postoperative complications.

Variables	Combined group(*n* = 39)	Ab Interno Trabeculotomy alone group(*n* = 44)	*P*-value
Visible hyphema, *n* (%)	9 (23.08)	19 (43.18)	0.053^a^
IOP spike, *n* (%)	5 (12.82)	17 (38.63)	0.005^a^
Duration of IOP spike (days)	6.0 (5.0, 10.0)	8.0 (7.0, 9.5)	0.385^b^
Onset within 1 week, *n* (%)	2 (5.13)	3 (6.82)	0.747^c^
Onset within 2 week, *n* (%)	3 (7.69)	12 (27.27)	0.021^a^
Peak IOP during spike (mmHg)	51.2 (45.6, 60.0)	52.7 (42.7, 60.0)	0.967^b^
Persistent IOP spike, *n* (%)	0 (0.00)	3 (6.82)	0.288^c^

IOP, intraocular pressure; ^a^Pearson chi-square test was used for comparisons of Visible hyphema, IOP spike, and Onset within 2 weeks; ^b^Mann–Whitney *U* test was applied for Duration and Peak IOP, with Z-values reported as the test statistic; ^c^Fisher's exact test was performed for Onset within 1 week and Persistent IOP spike (where expected frequencies were <5), and the exact *P*-value is reported directly.

## Discussion

This retrospective study demonstrated that, in patients with POAG, combined ab interno trabeculotomy and phacoemulsification achieved superior IOP reduction compared with ab interno trabeculotomy alone during the early postoperative follow-up period. However, at the 12-month follow-up, the difference in actual postoperative IOP between the two groups diminished and was no longer statistically significant. The combined procedure was associated with a better IOP-lowering effect and a lower incidence of a specific complication IOP spike to ab interno trabeculotomy alone. According to the American Academy of Ophthalmology, a reduction in IOP of 20%–30% from baseline is recommended, with the target adjusted based on the extent of visual field damage (7). In the Advanced Glaucoma Intervention Study, maintaining IOP below 18 mmHg was associated with a significant slowing of visual field defect progression over extended follow-up (8). It is also recommended that IOP be maintained below 18 mmHg in patients with mild to moderate glaucoma (9). In early glaucoma studies, every additional 1 mmHg reduction in IOPduring the first 3 months of treatment reduced the risk of disease progression by approximately 10% (10). Our treatment goals are to reduce IOP and the burden of glaucoma medications in patients. The core mechanism of ab interno trabeculotomy for POAG is to directly relieve the greatest site of resistance in the aqueous humor outflow pathway. Studies have demonstrated that the primary resistance to aqueous outflow originates from the trabecular meshwork, particularly the juxtacanalicular tissue. In POAG patients, this resistance is further increased due to extracellular matrix deposition within the trabecular meshwork, collapse and narrowing of Schlemm's canal, and degeneration of the inner wall endothelial cells (11). Ab interno trabeculotomy uses a specialized instrument to incise the full thickness of the trabecular meshwork and the inner wall of Schlemm's canal from the anterior chamber over approximately 120°, creating a direct channel that allows aqueous humor to bypass the high-resistance juxtacanalicular tissue and enter the lumen of Schlemm's canal. In addition, pressure recovery at the cut ends of Schlemm's canal following the incision may reduce tissue herniation into the orifices of collector channels and improve drainage function of the collector channels and aqueous veins (12). The additional IOP-lowering effect of the combined procedure may be attributable, in part, to the cataract extraction component. Possible mechanisms include the removal of lens-derived inflammatory factors and a stretching effect on the trabecular meshwork via expansion of the angle structures following lens removal. Furthermore, the reduction in prostaglandin and cytokine levels after phacoemulsification may facilitate improvement of the conventional outflow pathway. A key finding is that the IOP-lowering effect of phacoemulsification is positively correlated with the volume of infusion fluid used during surgery (13). Cataract extraction leads to sustained IOP reduction, and this delayed effect may be attributed to reduced iridolenticular contact and improved clearance of debris from the trabecular meshwork. The effect is most pronounced in eyes with exfoliation syndrome,where higher preoperative IOP is associated with a greater magnitude of IOP reduction after cataract surgery. The magnitude of IOP reduction is associated with the volume of infusion fluid used during the procedure. When phacoemulsification is combined with aspiration of aqueous outflow materials, the procedure has been reported to achieve a mean IOP reduction of 14 mmHg at 2 years (14). Based on these principles, active cleaning techniques, notably trabecular aspiration, have been developed to more effectively remove debris from the trabecular meshwork (15). Collectively, phacoemulsification does facilitate clearance of debris from the trabecular meshwork, and this clearance is achieved through the dual mechanisms of passive irrigation and active aspiration.

Both groups showed a reduction in the number of glaucoma medications, with no statistically significant difference. This reduction not only indicates improved IOPcontrol but also enhances patients’ quality of life. Given that many patients struggle with adherence to multiple daily instillations and that preservatives in antiglaucoma medications can worsen common age-related ocular surface disease, lowering the medication burden may improve long-term compliance and reduce ocular surface toxicity and discomfort.

In this study, both surgical procedures demonstrated favorable IOP reduction and postoperative glaucoma medication control, without serious or unexpected complications. The incidence of hyphema was significantly lower in the combined surgery group. Hyphema, the most common complication following ab interno trabeculotomy-type procedures, generally results from direct mechanical injury during surgery or reflux from the aqueous veins. The primary mechanism of reflux-related hyphema involves a reversed pressure gradient:when the inner wall of Schlemm's canal is incised, the anterior chamber pressure may transiently fall below the episcleral venous pressure, causing blood to reflux from the open ends of Schlemm's canal into the anterior chamber. In the combined group, the surgical sequence was ab interno trabeculotomy followed by cataract surgery, which may further reduce the risk of hyphema. Similar results have been reported in other comparable studies (16). When cataract surgery is performed after completion of the ab interno trabeculotomy, any blood that may have seeped into the anterior chamber can be more thoroughly removed, thus preventing postoperative hyphema. Moreover, because phacoemulsification may provoke a pronounced inflammatory response, performing the ab interno trabeculotomy first may avoid exposing the most vulnerable tissues to the strongest inflammatory stimulus, thereby lowering the risk of bleeding (17). In ab interno trabeculotomy alone, more pronounced local hemodynamic changes may increase the risk of blood reflux from the cut ends of Schlemm's canal. In the combined procedure, performing cataract surgery before ab interno trabeculotomy may result in a smoother IOP reduction, reducing reflux triggered by sudden pressure changes. Moreover, while both techniques include a final step to clear viscoelastic material and blood from the anterior chamber, the combined group uses the phacoemulsifier's irrigation/aspiration system, whereas the ab interno trabeculotomy-alone group uses a syringe. The irrigation/aspiration system provides more thorough clearance, which may further explain the reduced incidence of postoperative hyphema in the combined group.

The marked difference in the incidence of IOP spikes may be the most striking finding of this study. IOP spikes typically peak during specific postoperative periods, with time windows varying by procedure type. For ab interno trabeculotomy-type surgeries, IOP spikes usually occur within the first two postoperative weeks (mean time to diagnosis: 7.7 ± 6.5 days), most commonly within the first postoperative month, and have a mean duration of 4.9 ± 5.4 days (18). In a post-GATT study, IOP spike was associated with a significantly higher risk of surgical failure and has been identified as a predictor of surgical failure (19). The exact mechanism underlying IOP spikes remains unclear, but it is thought to be related to postoperative hyphema, postoperative anterior chamber inflammation, residual viscoelastic material, obstructive deposition of blood cells, inflammatory cells, and pigment within the trabecular meshwork, hyperreflective fibrous tissue covering the trabecular meshwork surface, use of postoperative topical corticosteroids, reattachment following postoperative cyclodialysis, scarring or closure of the aqueous outflow pathways, extensive formation of peripheral anterior synechiae involving more than three quadrants, and sudden changes in aqueous humor outflow patterns (20–22). Among the proposed mechanisms, IOP elevation due to residual viscoelastic material typically resolves spontaneously within 24–36 h; if elevated IOP is noted on postoperative day 1, a limited paracentesis may be performed, and release of a small amount of viscoelastic material confirms the diagnosis. For hyphema-induced IOP elevation, IOP usually decreases as the blood clears. In the few patients with persistent hyphema at two weeks postoperatively, anterior chamber washout may be performed, and a subsequent IOP reduction suggests that the IOP spike was caused by hyphema. In our cohort, all patients who experienced IOP spike had concurrent significant postoperative inflammation. Given that this factor was universally present across all cases, we were unable to statistically isolate the independent contribution of the inflammatory response to the IOP spikes. In the standalone ab interno trabeculotomy group, one patient developed a substantial hyphema, and the IOP returned to normal after anterior chamber washout; therefore, we attributed this IOP spike to the hyphema. For the other patients who experienced IOP spikes, the hyphema resolved spontaneously within one week postoperatively. However, the IOP spikes persisted even after the hyphema had completely resolved, which rules out hyphema as the primary cause in these cases. Also in the standalone trabeculotomy group, one patient underwent anterior chamber paracentesis in the early postoperative period, where retained viscoelastic material was clearly observed. The IOP normalized promptly after drainage, indicating that residual viscoelastic substance was the cause of the IOP spike in this specific case. Regarding the remaining IOP spike cases of unknown etiology, the spikes persisted after discontinuation of topical corticosteroids, and no recent adjustments had been made to the glaucoma medication regimens in any of these patients. These findings suggest that steroid response or medication changes were unlikely to be contributing factors.The lower rate of IOP spikes in the combined group may be attributable to the reduced hyphema rate. One retrospective study reported that the incidence of transient IOP elevation associated with hyphema was 0% in the combined cataract-ab interno trabeculotomy group vs. 16% in the ab interno trabeculotomy-alone group (23). Transient IOP elevation related to postoperative topical corticosteroid use usually resolves after steroid discontinuation. Cyclodialysis, a common complication after ab interno trabeculotomy-type procedures, typically resolves spontaneously. During cyclodialysis, aqueous may preferentially divert into the suprachoroidal space, causing a transiently low IOP; upon cyclodialysis reattachment, IOP returns to baseline. We hypothesize that a transient IOP elevation may occur in a minority of patients during reattachment, as the sudden loss of aqueous drainage into the suprachoroidal space may lead to an IOP rise. One study evaluating outcomes after ab interno trabeculotomy for POAG (without stratification by cataract surgery status) reported an overall IOP spike rate of 15.6%. A multicenter retrospective single-arm study of 81 patients reported a 5% IOP spike rate after combined ab interno trabeculotomy and phacoe mulsification (24). Patients with juvenile glaucoma may have a higher probability of postoperative IOP spikes following ab interno trabeculotomy-type procedures. One study on juvenile glaucoma reported a postoperative IOP spike rate as high as 74% (25). Our study offers new evidence demonstrating that the combined procedure substantially lowers the IOP spike rate. In a study of circumferential gonioscopy-assisted transluminal ab interno trabeculotomy that mainly enrolled patients with juvenile glaucoma and secondary open-angle glaucoma, early transient postoperative IOP elevation was found to be associated with the following angle-related factors: reattached trabecular meshwork remnants, iris processes in eyes with developmental glaucoma and iris anomalies, and peripheral anterior synechiae (26). We also hypothesize that the irrigation/aspiration function of the phacoemulsification handpiece during anterior chamber clearance in cataract surgery may simultaneously clean debris from the trabecular meshwork. In addition, the IOL may physically maintain anterior chamber depth and angle configuration, preventing Schlemm's canal collapse and collector channel closure that could otherwise occur after ab interno trabeculotomy alone. These factors may represent additional contributors to the reduced postoperative IOP spike rate.

This study has several limitations inherent to its retrospective design. In this retrospective cohort, the choice of surgical strategy was primarily determined by cataract severity, visual impairment, surgical feasibility, and patient preference. Patients who underwent ab interno trabeculotomy alone either had only mild cataracts without surgical indications or declined concurrent cataract extraction despite meeting the criteria. Consequently, the treatment allocation reflects real-world clinical decisions rather than randomized assignment, inevitably contributing to baseline imbalances between the two groups. These inherent selection biases should be carefully acknowledged when interpreting the comparative outcomes and represent a major limitation of this study. Second, the sample size was relatively small, and larger prospective studies are needed to confirm the differences in hyphema and IOP spike rates.Third, follow-up was limited to 12 months; longer follow-up (3–5 years) is necessary to assess the durability of the IOP-lowering effect and the risk of late-onset complications.Fourth,visual field analysis was not included as an endpoint, which would have enhanced clinical relevance.This was a single-center study with all surgeries performed by one senior surgeon, which may limit generalizability. Finally, because no preoperative washout was performed, the assessment of efficacy was made in the context of concomitant medication use, potentially confounded by the effects of IOP-lowering drugs. Nevertheless, this approach more closely reflects real-world clinical practice.

In this 12-month retrospective comparative study, combined ab interno trabeculotomy and phacoemulsification showed better clinical outcomes than ab interno trabeculotomy alone. The combined group had a significantly lower rate of IOP spikes. Both procedures were safe, with no serious adverse events. These results suggest that the combined procedure may offer advantages in terms of IOP control and a reduced risk of IOP spikes. However, because no statistically significant differences were observed in medication reduction, or surgical success rates between the two groups, and given the inherent selection bias, baseline imbalances, and retrospective nature of this study, the combined procedure cannot be definitively recommended as the preferred approach based on the current evidence. The present findings should be interpreted as hypothesis-generating rather than practice-changing, and larger, longer-term prospective randomized controlled trials including visual field endpoints are needed to confirm these results.

## Data Availability

The raw data supporting the conclusions of this article will be made available by the authors, without undue reservation.

## References

[1] Tham YC Li X Wong TY Quigley HA Aung T Cheng CY. Global prevalence of glaucoma and projections of glaucoma burden through 2040. Ophthalmology. (2014) 121(11):2081–90. 10.1016/j.ophtha.2014.05.013 24974815

[2] Gedde SJ Vinod K Wright MM Muir KW Lind JT Chen PP. Primary open-angle glaucoma preferred practice pattern®. Ophthalmology. (2021) 128(1):P71–P150. 10.1016/j.ophtha.2020.10.022 34933745

[3] Schuster AK Wagner FM Pfeiffer N Hoffmann EM. Risk factors for open-angle glaucoma and recommendations for glaucoma screening. Ophthalmologe. (2021) 118(Suppl 2):145–52. English. 10.1007/s00347-021-01378-5 33881589

[4] Goel M Picciani RG Lee RK Bhattacharya SK. Aqueous humor dynamics: a review. Open Ophthalmol J. (2010) 4:52–9. 10.2174/1874364101004010052 21293732 PMC3032230

[5] Casson RJ Chidlow G Wood JP Crowston JG Goldberg I. Definition of glaucoma: clinical and experimental concepts. Clin Exp Ophthalmol. (2012) 40(4):341–9. 10.1111/j.1442-9071.2012.02773.x 22356435

[6] Jonas JB Aung T Bourne RR Bron AM Ritch R Panda-Jonas S. Glaucoma. Lancet. (2017) 390(10108):2183–93. 10.1016/S0140-6736(17)31469-1 28577860

[7] Gedde SJ Bowden EC Challa P Vinod K Kolomeyer NN Chopra V. Primary open-angle glaucoma preferred practice pattern®. Ophthalmology. (2026) 133(4):P1–P103. 10.1016/j.ophtha.2025.12.029 41665583

[8] The Advanced Glaucoma Intervention Study (AGIS): 7. The advanced glaucoma intervention study (AGIS): 7. The relationship between control of intraocular pressure and visual field deterioration. Am J Ophthalmol. (2000) 130(4):429–40. 10.1016/s0002-9394(00)00538-9 11024415

[9] Sihota R Angmo D Ramaswamy D Dada T. Simplifying “target” intraocular pressure for different stages of primary open-angle glaucoma and primary angle-closure glaucoma. Indian J Ophthalmol. (2018) 66(4):495–505. 10.4103/ijo.IJO_1130_17 29582808 PMC5892050

[10] Leske MC Heijl A Hussein M Bengtsson B Hyman L Komaroff E. Factors for glaucoma progression and the effect of treatment: the early manifest glaucoma trial. Arch Ophthalmol. (2003) 121(1):48–56. 10.1001/archopht.121.1.48 12523884

[11] Koliakos GG Konstas AG Schlötzer-Schrehardt U Bufidis T Georgiadis N Ringvold A. Ascorbic acid concentration is reduced in the aqueous humor of patients with exfoliation syndrome. Am J Ophthalmol. (2002) 134(6):879–83. 10.1016/s0002-9394(02)01797-x 12470757

[12] Shingleton BJ Laul A Nagao K Wolff B O'Donoghue M Eagan E. Effect of phacoemulsification on intraocular pressure in eyes with pseudoexfoliation. J Cataract Refract Surg. (2008) 34(11):1834–41. 10.1016/j.jcrs.2008.07.025 19006727

[13] Damji KF Konstas AG Liebmann JM Hodge WG Ziakas NG Giannikakis S. Intraocular pressure following phacoemulsification in patients with and without exfoliation syndrome: a 2 year prospective study. Br J Ophthalmol. (2006) 90(8):1014–8. 10.1136/bjo.2006.091447 16672324 PMC1857224

[14] Jacobi PC Dietlein TS Krieglstein GK. Bimanual trabecular aspiration in pseudoexfoliation glaucoma. Ophthalmology. (1998) 105(5):886–94. 10.1016/S0161-6420(98)95032-1 9593393

[15] Jacobi PC Krieglstein GK. Trabecular aspiration: a new surgical approach to improve trabecular facility in pseudoexfoliation glaucoma. Int Ophthalmol. (1994) 18(3):153–7. 10.1007/BF00915964 7852021

[16] Zhang S He W Lu P Xu F Zhong H Zhong S. Optimizing the sequential approach of combined phacoemulsification and gonioscopy-assisted transluminal trabeculotomy (GATT) in primary open-angle glaucoma. BMC Ophthalmol. (2025) 25(1):256. 10.1186/s12886-025-04097-6 40296008 PMC12036275

[17] Miura Y Fukuda K. Comparison of different procedures in a combination of ab interno microhook trabeculotomy and cataract surgery. J Clin Med. (2022) 11(3):738. 10.3390/jcm11030738 35160197 PMC8837028

[18] Sakamoto T Nisiwaki H. Factors associated with 1-year outcomes and transient intraocular pressure elevation in minimally invasive glaucoma surgery using kahook dual blades. Sci Rep. (2023) 13(1):15206. 10.1038/s41598-023-42575-3 37710010 PMC10502046

[19] Naftali Ben Haim L Yehezkeli V Abergel Hollander E Dar N Sharon T Belkin A. Intraocular pressure spikes after gonioscopy-assisted transluminal trabeculotomy (GATT). Graefes Arch Clin Exp Ophthalmol. (2024) 262(3):927–35. 10.1007/s00417-023-06265-0 37843563

[20] Uzel MM Koçer AM Aksoy BE Hondur G. Intraocular pressure spikes after trabecular meshwork strip peeling during hemi-GATT. Graefes Arch Clin Exp Ophthalmol. (2026) 264:2235–43. 10.1007/s00417-026-07182-8 41807822 PMC13342195

[21] Shalaby WS Chang SL Yu J Horan T Shah S Hallaj S. Impact of postoperative intraocular pressure spikes on outcomes following goniotomy: a comparative study of surgical techniques. J Curr Glaucoma Pract. (2025) 19(4):186–98. 10.5005/jp-journals-10078-1498 41523173 PMC12780354

[22] Taskin G Alagoz N Cakir I Altan C Akgun GG Yasar T. Insights into intraocular pressure spikes following gonioscopy-assisted transluminal trabeculotomy (GATT): timing, predictors and clinical outcomes. Jpn J Ophthalmol. (2026) 70(1):199–209. 10.1007/s10384-025-01273-5 40900384

[23] Inatani M Tanihara H Muto T Honjo M Okazaki K Kido N. Transient intraocular pressure elevation after trabeculotomy and its occurrence with phacoemulsification and intraocular Lens implantation. Jpn J Ophthalmol. (2001) 45(3):288–92. 10.1016/s0021-5155(01)00322-7 11369380

[24] Hirsch L Cotliar J Vold S Selvadurai D Campbell A Ferreira G. Canaloplasty and trabeculotomy ab interno with the OMNI system combined with cataract surgery in open-angle glaucoma: 12-month outcomes from the ROMEO study. J Cataract Refract Surg. (2021) 47(7):907–15. 10.1097/j.jcrs.0000000000000552 33315733

[25] Shi Y Wang H Oatts JT Xin C Yin P Zhang L. A prospective study of intraocular pressure spike and failure after gonioscopy-assisted transluminal trabeculotomy in juvenile open-angle glaucoma. Am J Ophthalmol. (2022) 236:79–88. 10.1016/j.ajo.2021.10.009 34695398

[26] Rao A Khan SM Mukherjee S. Causes of immediate and early IOP spikes after circumferential gonioscopy-assisted transluminal ab interno trabeculotomy using ASOCT. Clin Ophthalmol. (2023) 17:313–20. 10.2147/OPTH.S397816 36711260 PMC9879041

